# Olfactory and gustatory perception in Brazilian PKU patients: A cross‐sectional study

**DOI:** 10.1002/jmd2.12445

**Published:** 2024-08-29

**Authors:** Tássia Tonon, Chenia Martinez, Tatiele Nalin, Soraia Poloni, Otávio Bejzman Piltcher, François Maillot, Bianca Fasolo Franceschetto, Ida Vanessa D. Schwartz

**Affiliations:** ^1^ Graduate Program in Medicine: Medical Sciences Universidade Federal do Rio Grande do Sul Porto Alegre Brazil; ^2^ Ultragenyx Brasil Farmacêutica Ltda São Paulo Brazil; ^3^ Nutrition and Dietetics Service Hospital de Clínicas de Porto Alegre Porto Alegre Brazil; ^4^ Department of Ophthalmology and Otolaryngology Universidade Federal do Rio Grande do Sul Porto Alegre Brazil; ^5^ Internal Medicine Department, Reference Center for Inherited Disease University of Tours Tours France; ^6^ Medical Genetics Service Hospital de Clínicas de Porto Alegre Porto Alegre Brazil; ^7^ Department of Genetics Universidade Federal do Rio Grande do Sul Porto Alegre Brazil

**Keywords:** gustation, inborn errors of metabolism, olfaction, phenylketonuria

## Abstract

Patients with phenylketonuria (PKU) have a highly restrictive diet, which involves restriction of phenylalanine (Phe) intake and daily use of Phe‐free metabolic formula. However, little is known about the potential impact of this diet on chemical senses. The present study aimed to evaluate the olfactory and gustatory perceptions of patients with PKU. A cross‐sectional controlled study which included patients with PKU on dietary treatment and healthy controls was performed. Olfactory perception was assessed using the 12‐item *Sniffin’ Sticks* test, and taste perception using the *Taste Strips* test. Twenty‐five patients (mean age 19.3 ± 4.7 years; 13 females) and 25 controls (mean age 19.9 ± 4.9 years, *p* = 0.676; 13 females) were included. The mean age at treatment onset was 52.8 ± 29.7 days. The mean scores for olfactory and gustatory perceptions, and for bitter and salty flavors, were lower in patients than in controls (*p* = 0.039, *p* = 0.004, *p* = 0.008, and *p* = 0.020, respectively). Among patients, Phe levels at diagnosis correlated negatively with bitter taste (*r* = −0.493, *p* = 0.006). The lower olfaction and gustation scores found in patients may be related to understimulation caused by the highly restrictive PKU diet and the deprivation of flavors from breast milk.

## 1 INTRODUCTION

Phenylketonuria (PKU) is an inborn error of amino acid metabolism caused by deficient activity of phenylalanine hydroxylase, which catalyzes conversion of phenylalanine (Phe) into tyrosine. Lifelong treatment is recommended,^1^ mostly relying on nutritional management through a highly Phe‐restricted diet and supplemented with a Phe‐free mixture of synthetic amino acids (known as the metabolic formula), which serves as the main protein and micronutrient source of every PKU patient's diet.^2^ The goal of treatment is to maintain plasma Phe concentrations within recommended values, to support adequate growth as well as optimal physical and neurological development.^3^ A major challenge in PKU is the need for constant adherence to the Phe diet, which is highly restrictive^4^ and associated with the use of a metabolic formula that is made up of amino acids (except Phe), which provides a sour and bitter taste, in addition to having unpleasant volatile components, such as sulfur found in cysteine.^5^

To date, little is known about the impact of these dietary restrictions on the chemical senses of these patients. The primary function of the senses of taste and smell is the ability to distinguish between foods that are harmful (aversive), which can even be deadly, and those that provide pleasure and nutrition (appetitive).^6^, ^7^ The complex interaction between taste, smell, and trigeminal sense (touch and temperature) results in this flavor that can be aversive or appetitive. The flavor result can be sweet, umami, bitter, salty, or sour, which are the five primary gustatory sensory qualities, among others.^7^ In the past, some authors believed that in PKU, the onset of metabolic formula intake had a major influence on the development of dietary preferences and might actually entrain a preference for bitter and sour flavors. Later, it was found that children with PKU aged ≥4 years preferred savory foods (vegetables) more than control children, but they did not prefer bitter‐tasting foods.^2^

Children who receive protein hydrolysate formulas, the taste of which resembles PKU metabolic formula, are more likely to prefer salty, bitter, and sour foods, when compared to children who are fed breastmilk or sweeter milk‐based formulas.^8^ Thus, it is possible that patients with PKU have different olfactory and gustatory perceptions because of the intake of metabolic formula and deprivation of several foods due to a Phe‐restricted diet in the first months of life. Within this context, the present study aimed to evaluate the olfactory and gustatory perceptions of Brazilian patients with PKU and ascertain whether they are associated with disease markers.

## 2 METHODS

This exploratory cross‐sectional controlled study enrolled patients who are followed at the Outpatient Metabolic Disorders Clinic of the Medical Genetics Service at Hospital de Clínicas of Porto Alegre, a hospital in Southern Brazil. The study protocol was approved by the local Institutional Review Board (150072). The inclusion criteria were age ≥9 years (deemed as the minimum for understanding the applied tests), current nutritional management with Phe‐free metabolic formula, and absence of any intellectual impairment assessed by clinical criteria. Twenty‐five patients agreed to participate. The sample also included 25 healthy controls (free of any metabolic diseases, absence of any intellectual impairment, and not taking any medication that may change the taste and smell), matched for age and sex. Data were collected from 2016 to 2018.

Clinical information, such as mean plasma Phe levels at diagnosis and during the year prior to study inclusion, was obtained by means of chart review. Good metabolic control was defined as in the European guidelines^1^; for patients <12 years of age, a mean Phe level < 360 μmol/L on at least three measurements obtained in the 12 months prior to study enrollment was considered satisfactory. For patients over 12 years of age, mean Phe levels <600 μmol/L were required. Data on smoking and history of breastfeeding were also collected through a specific questionnaire.

Weight and height were used to calculate the body mass index (BMI), which was expressed as the *z*‐score for age and sex; patients were then classified as underweight, normal weight, overweight, or obese, according to the 2007 World Health Organization criteria.

### 2.1 Procedure

Olfactory perception was evaluated through the 12‐item *Sniffin’ Sticks* test (Burghart, Wedel, Germany), which is based on the identification of scents on pen‐like devices saturated with odorants.^9^ Briefly, for each of the 12 *Sniffin’ Sticks*, participants were asked to choose the correct answer from a list of four words [for an example: option (1) orange; option (2) strawberry; option (3) blackberry; and option (4) pineapple], among which was that corresponding to the actual odorant. The sum score ranges from 0 to12, and classified patients as having normal olfactory perception (≥10), hyposmia (decreased olfactory perception, ≥6–9), or anosmia (total loss of olfactory perception, ≤5). Patients who screened positive for hyposmia or anosmia were invited to undergo an otorhinolaryngological evaluation to ascertain whether any anatomical abnormalities in the airways might have affected the test result. The otorhinolaryngology specialist who performed this evaluation also asked a question to assess patients' subjective olfactory perception: “What do you think of your olfactory perception?”

Gustatory perception was evaluated through the *Taste Strips* test (Burghart, Wedel, Germany). Currently, five general categories called primary sensations of taste are known, namely: sour, salty, bitter, sweet, and umami. However, this study focused on the gustatory perception of four qualities: sour, salty, bitter, and sweet. The kit consists of 18 strips of filter paper; 16 impregnated with four different concentrations of flavors corresponding to the four basic tastes (sweet, bitter, sour, and salty), as well as two control strips with no added flavors. The strips were placed onto the anterior third of the right and left sides of the tongue, as well as the midline region. When switching strips, participants were instructed to rinse their mouths out with water. Strips were applied as per the manufacturer's instructions (from the lowest to the highest concentration and alternating flavors). The sum score of all stimuli ranges from 0 to 16 and is classified as normogeusia (normal taste perception, ≥10–16) or hypogeusia (decreased taste perception, ≤9). Scores for each specific taste range from 0 to 4 points.

Statistical analyses were carried out in PASW Statistics, Version 18.0 (SPSS Inc., Chicago, IL). Descriptive analysis consisted of absolute frequencies. Continuous variables were expressed as mean and standard deviation. Student's *t*‐test and the chi‐square test for paired or independent samples were used, and Pearson correlations were calculated. The level of significance was set at 5%.

## 3 RESULTS

Overall, 25 patients (13 females) and 25 controls (13 females) participated in the study. The mean (SD) age of patients was 19.3 ± 4.7 years (range: 9.3–30.6), while that of controls was 19.9 ± 4.9 years (range: 8.4–32.9, *p* = 0.676). The mean Phe level was 1360.2 ± 671.3 μmol/L (range: 344.9–2946.4) at diagnosis and 710.5 ± 346.4 μmol/L (range: 215.1–1408.9) during the 12 months prior to the study. The mean age at treatment onset was 52.8 ± 29.7 days (range: 11–134). Clinical and demographic data are presented in Table 1. We emphasize that patient number 25 in the table presented a Phe level of 344.9 μmol/L at diagnosis, which may suggest mild hyperphenylalaninemia. However, the patient is diagnosed with PKU, as their Phe levels reached 1709.12 μmol/L without any treatment (diet or metabolic formula).

**TABLE 1:** Demographic and clinical characteristics of patients with phenylketonuria (*n* = 25).

Pt	Sex	Age	Phe level at diagnosis (μmol/L)	Mean Phe level in the 12 months prior to study (μmol/L)	BF	SST	Otorhinolaryngological evaluation	TS	SW	SO	SA	BI	Smoker	Good metabolic control
1	F	15.0	1996.5	555.6	No	7	Yes*	8	4	0	3	1	No	Yes
2	M	14.9	1022.5	470.2	No	7	Yes	14	3	4	3	4	No	Yes
3	M	15.8	1845.3	412.2	No	9	No	10	4	3	0	3	No	Yes
4	F	16.2	1070.9	695.7	No	6	Yes	6	3	2	1	0	No	No
5	F	24.3	1633.5	277.5	No	11	–	12	4	3	3	2	No	Yes
6	M	20.0	2051.0	1194.2	No	10	–	8	3	2	3	0	No	No
7	M	16.9	2268.8	933.1	No	10	–	11	4	3	2	2	No	No
8	F	19.2	1724.3	627.5	No	9	–	8	3	2	2	1	No	No
9	M	26.2	2946.4	756.3	No	8	Yes	10	3	3	4	0	No	No
10	F	20.2	1923.9	1172.8	No	9	No	9	3	2	2	2	No	No
11	F	18.5	901.5	1072.1	No	11	–	12	4	2	3	3	No	No
12	F	9.3	1784.8	661.7	No	9	No	10	4	2	4	0	No	No
13	F	18.4	605.0	520.1	No	9	No	9	4	1	3	1	No	Yes
14	M	15.8	871.2	651.1	No	11	–	5	2	2	0	1	No	No
15	M	17.3	1681.9	339.2	No	7	Yes*	8	3	1	3	1	No	Yes
16	F	21.6	901.5	281.2	No	11	–	12	4	3	4	1	No	Yes
17	M	16.4	490.1	380.9	No	9	No	9	4	3	0	2	No	Yes
18	M	27.7	2099.4	924.8	No	12	–	7	3	1	3	0	No	No
19	M	21.8	738.1	1324.1	No	10	–	10	4	3	1	2	No	No
20	F	17.6	1179.8	699.6	No	10	–	13	3	3	3	4	No	No
21	M	21.7	847.0	871.1	No	11	–	9	3	1	3	2	No	No
22	F	16.6	405.4	324.0	No	11	–	15	3	4	4	4	No	Yes
23	F	25.2	1028.5	995.7	No	8	No	11	3	3	2	3	No	No
24	M	30.6	1645.6	1408.9	No	8	No	10	3	3	1	3	No	No
25	F	17.0	344.9	215.1	No	11	–	14	4	3	3	4	No	Yes

*Note*: Pt, patient; F, female, M, male; age in years; BF: breastfeeding: none of the 25 patients included in the study has received exclusive breastfeeding until age 6 months; SST: *Sniffin’ Sticks* Test (varies from 0 to 12); Otorhinolaryngological evaluation, clinical evaluation + flexible rhinoscopy (*two cases reported reduced olfaction); Good metabolic control: patients <13 years old: mean Phe <360 μmol/L in the 12 months prior to the study; patients ≤13 years old: mean Phe <600 μmol/L. All patients were on diet+metabolic formula.

Abbreviations: BI, bitter; SA, salt; SO, sour; SW, sweet; TS, Taste Strips.

Due to treatment of PKU and introduction of the metabolic formula, none of the 25 patients included in the study has received exclusive breastfeeding until age 6 months (Table 1). Conversely, 21 controls had received exclusive breastfeeding up to age 6 months (*p* < 0.001). At the time of the study, 17 patients (68%) used sugar, maltodextrin, or other flavoring agents to mask the taste of the metabolic formula.

Regarding olfactory perception, the mean score of PKU patients was significantly lower (9.3 ± 1.6) than that of controls (10.3 ± 1.6, *p* = 0.039). Overall, 13 (52%) patients had mean olfaction scores below the cutoff point for hyposmia, versus only 7 (28%) of controls (*p* = 0.039). No patient or control had scores indicative of anosmia.

Regarding gustatory perception, the mean score of patients with PKU was significantly lower (10.0 ± 2.5) than that of controls (11.9 ± 2.0, *p* = 0.004). Among patients, 11 (44%) had mean gustatory scores below the cutoff point for hypogeusia, versus only 3 (12%) of controls. Table 2 presents the mean scores of patients and controls for each of the flavors tested. Scores for bitter and salty flavors were significantly lower in patients than in controls (*p* = 0.008 and *p* = 0.020, respectively). Furthermore, Phe levels at diagnosis correlated with scores for bitter (*r* = −0.493, *p* = 0.006), but not with any other taste. No correlation was found between the bitter scores and the mean Phe level in the 12 months prior to study (*r* = −0.113, *p* = 0.295), or with any other taste.

**TABLE 2:** “Taste Strips” scores for sweet, sour, salty, and bitter flavor and the *Sniffin's Sticks* test in patients with phenylketonuria and healthy controls.

	Patients (*n* = 25)	Controls (*n* = 25)	p*
Sweet	3.40 ± 0.5	3.48 ± 0.5	0.629
Sour	2.36 ± 0.9	2.52 ± 0.6	0.505
Salty	2.40 ± 1.2	3.12 ± 0.7	**0.020**
Bitter	1.84 ± 1.3	2.84 ± 1.1	**0.008**
SST	9.36 ± 1.6	10.32 ± 1.6	**0.039**

*Note*: p*: *t*‐test. For Taste Strips: scores for each specific taste range from 0 to 4 point; SST: *Sniffin's Sticks* Test: scores range from 0 to 12; Mean scores and standard deviations are reported.

No significant correlation was observed between presence of hyposmia and hypogeusia and Phe levels at diagnosis (*r* = −0.237; *p* = 0.127 and *r* = −0.310; *p* = 0.065, respectively), as well as mean Phe levels during the 12 months preceding the study (*r* = −0.005; *p* = 0.491 and *r* = −0.267; *p* = 0.099, respectively). Furthermore, no correlation was observed between the olfactory and gustatory perceptions test in the patient group (*r* = 0.229, *p* = 0.272) and in the control group (*r* = −0.133, *p* = 0.263).

Five of the 13 patients who screened positive for hyposmia underwent clinical examination and flexible rhinoscopy by an otorhinolaryngologists. None of these patients had any abnormalities which might impair smell perception, such as septal deviation. Two patients explicitly complained of reduced scent perception (Table 1).

## 4 DISCUSSION

The present study was the first to evaluate the olfactory and gustatory perceptions of patients with PKU and their potential association with disease markers. Our novel findings possibly indicate a significant prevalence of hyposmia and hypogeusia in patients with PKU when compared to healthy controls. Furthermore, we observed lower perception scores for bitter and salty tastes among patients as compared to controls. High Phe levels at diagnosis are usually associated with major consumption of metabolic formula during life, and this may interfere in the perceptions of bitter taste later in life.

According to the literature, early experiences with scent, which occur since the intrauterine life, may influence food acceptance after breastfeeding, as the placental aroma may change according to the mother's dietary choices.^10^ Taste learning continues after birth because of exposure to infant formulas and/or breast milk, followed by the introduction of solid foods. Unlike infant formula, breastfeeding provides a wealth of variation in gustatory experiences. Children who receive bitter protein hydrolysates during childhood are more likely to prefer salty, bitter, and sour foods than children fed sweeter milk‐based formulas.^8^ In our sample of PKU patients, use of metabolic formula, a substantial reduction or even absence of breastfeeding probably altered olfactory and gustatory perceptions.

Another study conducted by Martinez and colleagues (2018)^11^ has assessed the impact of a highly restrictive diet on taste and smell in patients with inborn errors of metabolism. In that study, 22 patients with hepatic glycogen storage diseases (GSD), aged 11 years or older, were evaluated. Of these, 40.9% screened positive for hyposmia and 18.2% for hypogeusia. The greatest difficulty experienced by patients was in recognizing sour taste, a fact possibly explained by understimulation because of the lack of fruit (one of the main sources of citrus flavor) in their diet. The mainstay of GSD treatment is nutritional, consisting of a diet free of sugars (fructose, maltose, glucose, lactose, and galactose), supplemented with uncooked cornstarch several times daily, which typically accounts for over 50% of the daily energy requirements.^12^, ^13^ This indicates that both early exposure and reduced exposure to a particular flavor can influence taste and olfactory perception, potentially increasing tolerance to the flavor or making it more difficult to identify.

Our study corroborates the findings of Martinez et al.^11^ insofar as it shows that olfactory and gustatory perception can be blunted by a restrictive diet which fails to provide appropriate stimuli of distinct flavors. Further evidence of this is provided by our thorough analysis of the chemical senses through otorhinolaryngological evaluation in PKU patients who screened positive for hyposmia, which did not show any anatomical defects that might interfere with normal taste and smell sensation.

The PKU metabolic formula is considered unpalatable to nonexposed individuals, that is, persons without PKU.^8^ However, a study by Owada et al,^4^ found that children with PKU preferred the “unpleasant” taste of the conventional metabolic formula to that of a new formulation which was considered more palatable by individuals who did not have PKU. In Brazil, only unflavored amino acid‐based formulas are available, and patients have no power in the process of selecting which brand of metabolic formula will be provided. Additionally, alternative treatments such as glycomacropeptide and large neutral amino acids are not available. The flavor of the provided formula often limits not only palatability but also treatment adherence, by forcing parents or patients to add excessive amounts of sugar, artificial sweeteners, or other flavorings to mask the unpleasant taste. According to Evans et al,^2^ children with PKU prefer sweet tastes when compared to healthy control children; consequently, intake of sugar‐sweetened foods, snacks, and beverages, such as soft drinks, ends up being higher in this population.

According to Guyton and Hall,^6^ humans are much more sensitive to bitter taste than to any other taste—a fact justified by the bitter flavor of many life‐threatening toxins. We believe that the decreased perception of the bitter taste exhibited in our study in patients chronically exposed to the metabolic formula is due to the phenomenon known as sensory‐specific satiety,^14^ which makes subjects less responsive to a flavor to which they are extensively exposed. Further studies are needed, maybe the lack of taste imprinting would lead to lower perception of the flavors to which children were most strongly exposed shortly after birth.

Although the literature shows that children with PKU are more likely to prefer sweet tastes than control children, there is evidence that they also prefer salty/savory foods, especially vegetables; and this may also be associated with use of the metabolic formula.^2^ Again, reduced perception of taste (in this case, savory) is associated with “sensory‐specific satiety,” which makes subjects less responsive to a flavor to which they are repeatedly exposed.

Reduced olfactory and gustatory perception may also be associated with the presence of feeding difficulties, which have been reported in the literature since MacDonald et al,^15^ According to Evans et al,^2^ a cohort of 35 children with PKU was more apprehensive about attempting to eat new, unknown foods than a matched cohort of 35 control children. Tonon et al,^16^ also found that food neophobia is frequent in Brazilian patients with PKU (*n* = 25 patients compared to 25 healthy controls, same sample as the one in the present study). This is consistent with the findings of Martinez et al,^11^ who noted that the gustatory, olfactory, and tactile sensory experiences of childhood (including exposure to different textures and thicknesses) play an important role in the promotion of adequate, enjoyable eating habits. In their study, Martinez et al,^11^ found that reduced olfactory perception was associated with food selectivity.

We believe sensory evaluation should be part of the routine assessment of patients with PKU experiencing eating difficulties to detect potential changes in the chemical senses caused by strict dietary treatment. Consequences of these changes may modify patients' future dietary choices and exacerbate nutritional and behavioral diseases, such as obesity and eating disorders.^17^ In addition, the impact of alternative treatments that alleviate dietary restrictions (such as BH_4_ and pegvaliase) or improvements in the protein substitute's palatability (such as glycomacropeptide) on olfactory and gustatory modulation will contribute to the understanding of the effect of PKU diet on chemical senses.

In conclusion, our findings suggest a significant prevalence of reduced olfactory and gustatory perception in Brazilian patients with PKU. We believe this occurs because of a daily intake of a considerable amount of amino acids formula and a substantial reduction or even absence of breastfeeding. These alterations might contribute to the development of feeding difficulties previously reported in PKU patients.

## AUTHOR CONTRIBUTIONS

Tássia Tonon: planning, data collection, database, analysis and writing, and review of manuscript. Chenia Martinez: planning and analysis and review of manuscript. Tatiele Nalin: analysis and writing and review of manuscript. Soraia Poloni: analysis and writing and review of manuscript. Otávio Bejzman Piltcher: data collection and writing and review of manuscript. François Maillot: writing and review of manuscript. Bianca Fasolo Franceschetto: writing and review of manuscript. Ida Vanessa D Schwartz: planning, database, analysis, writing, and review of manuscript. All authors contributed substantially to the study and approved the final version of the manuscript.

## CONFLICT OF INTEREST STATEMENT

Tássia Tonon, Chenia Martinez, Tatiele Nalin, Soraia Poloni, Otávio Bejzman Piltcher, François Maillot, Bianca Fasolo Franceschetto, and Ida Vanessa D Schwartzdatabase declare that they have no conflict of interest.

## ETHICAL STATEMENT

All procedures followed were in accordance with the ethical standards of the responsible committee on human experimentation (institutional and national) and with the Helsinki Declaration of 2013. The study protocol was approved by the local Institutional Review Board (150072).

## PATIENT CONSENT

All individuals who participated in this study agreed and signed the informed consent form in accordance with the rules and procedures of the local ethics council.

## ANIMAL STUDIES

This article does not contain any studies with animal subjects performed by the any of the authors.

## Data Availability

We confirm that the data for this work are available in the research group's database and can be requested by contacting the corresponding author.
